# Supplementary material for: Identification of Novel Micropeptides Derived from Hepatocellular Carcinoma-Specific Long Noncoding RNA
Source: Int J Mol Sci. 2021 Dec 21;23(1):58. doi: 10.3390/ijms23010058 (PMC8744898; doi:10.3390/ijms23010058)
Supplement: Supplementary file 1 [file ijms-23-00058-s001.zip › ijms-1503570-supplementary.pdf]

Table S1. Expression level of twelve HCC-specific lincRNAs in HepG2 and normal tissues (Fig. 1A).

	Expression levels in organs (FPKM)																				
**Transcript ID**	Liver	HepG2	adipose	adrenal	brain	brain_R	breast	colon	foreskin_R	heart	kidney	lung	lymphNode	ovary	placenta_R	prostate	skeletal Muscle	testis	testis_R	thyroid	leukocyte
NONHSAT226968.1	0	104.918	0	0	0	0	0	0	0	0	0	0	0	0	0	0	0	0	0	0	0
NONHSAT183029.1	0	75.7612	0	0	0	0	0	0	0	0	0	0	0	0	0	0	0	0	0	0	0
NONHSAT013026.2	0	61.2921	0	0	0	0	0	0	0	0	0	0	0	0	0	0	0	0	0	0	0
NONHSAT204527.1	0	45.6999	0	0	0	0	0	0	0	0	0	0	0	0	0	0	0	0	0	0	0
NONHSAT250607.1	0	39.3399	0	0	0	0	0	0	0	0	0	0	0	0	0	0	0	0	0	0	0
NONHSAT258915.1	0	38.9182	0	0	0	0	0	0	0	0	0	0	0	0	0	0	0	0	0	0	0
NONHSAT142412.2	0	30.5447	0	0	0	0	0	0	0	0	0	0	0	0	0	0	0	0	0	0	0
NONHSAT223630.1	0	25.1423	0	0	0	0	0	0	0	0	0	0	0	0	0	0	0	0	0	0	0
NONHSAT075615.2	0	16.5705	0	0	0	0	0	0	0	0	0	0	0	0	0	0	0	0	0	0	0
NONHSAT115455.2	0	14.9831	0	0	0	0	0	0	0	0	0	0	0	0	0	0	0	0	0	0	0
NONHSAT168790.1	0	13.7239	0	0	0	0	0	0	0	0	0	0	0	0	0	0	0	0	0	0	0
NONHSAT185209.1	0	10.1151	0	0	0	0	0	0	0	0	0	0	0	0	0	0	0	0	0	0	0

Table S2. Primer table.

Gene	Forward primer	Reverse primer
Gapdh	TGTTGCCATCAATGACCCCTT	CTCCACGACGTACTCAGCG
NONHSAT226968.1	TTTTAGACATTGTATGAGGCAGCATTGT	TAGACTCGCATCTCAAGATCCGA
NONHSAT013026.2	CTTGGGGCAGATGGCTTCCTG	ATGAGGCTTAGGGAGAAGGCT
NONHSAT250607.1	GGCCATGGAGAGAGAACACC	ATTTCCTCCACCGACAGAGC
NONHSAT115455.2	TGCGTGCTGTTTATTGGAGC	TGTCCCAAAAGGAGGACCTG
NONHSAT168790.1	CCCACTCAGTTCCACTTCCC	TCCGGAGCCTGAGACTTACA

Table S3. Potential phosphorylation sites of Linc013026-68AA.

	**Position in Linc013026-68AA**	**Sequence in Linc013026-68AA**	**Corresponding motif described in the literature (phosphorylated residues in red)**	**Features of motif described in the literature**	**Link to original article describing the motif**
**Tyrosin phosphorylation**	20-23	PEYS	X[E/D]pYX	EGFR kinase substrate motif	[1]
	21-22	EY	[E/D/Y]pY	TC-PTP phosphatase substrate motif	[2]
	22-23	YS	pY[A/G/S/T/E/D]	Src kinase substrate motif	[3]
**Serine and Threonine phosphorylation**	15-20	LSWLRP	X[pS/pT]XXX[A/P/S/T]	G protein-coupled receptor kinase 1 substrate motif	[4]
	22-24	YSQ	XpSQ	DNA dependent Protein kinase substrate motif	[5]
	23-24	SQ	pSQ	ATM kinase substrate motif	[3]
	23-25	SQT	pSX[E/**pS*/pT***]	Casein Kinase II substrate motif	[6]
	27-30	EIDT	[E/D]XX[pS/pT]	Casein Kinase I substrate motif	[7]

	29-33	DTWCQ	[E/D][pS/pT]XXX	b-Adrenergic Receptor kinase substrate motif	[4]
	37-40	EPPS	[E/D]XX[pS/pT]	Casein Kinase I substrate motif	[7]
	39-41	PSW	P[pS/pT]X	DNA dependent Protein kinase substrate motif	[8]
	44-47	DSLS	[E/D]XX[pS/pT]	Casein Kinase I substrate motif	[7]
	44-48	DSLSV	[E/D][pS/pT]XXX	b-Adrenergic Receptor kinase substrate motif	[4]
	45-47	SLS	pSX[E/**pS*/pT***]	Casein Kinase II substrate motif	[6]
	65-68	QPSP	XXpSP	GSK-3, ERK1, ERK2, CDK5 substrate motif	[9]
	66-68	PSP	X[pS/pT]P	GSK-3, ERK1, ERK2, CDK5 substrate motif	[9]
	66-68	PSP	P[pS/pT]X	DNA dependent Protein kinase substrate motif	[5]
	67-68	SP	pSP	ERK1, ERK2 Kinase substrate motif	[9]

* indicates the residue that has to phosphorylated already for the enzyme to recognize the motif

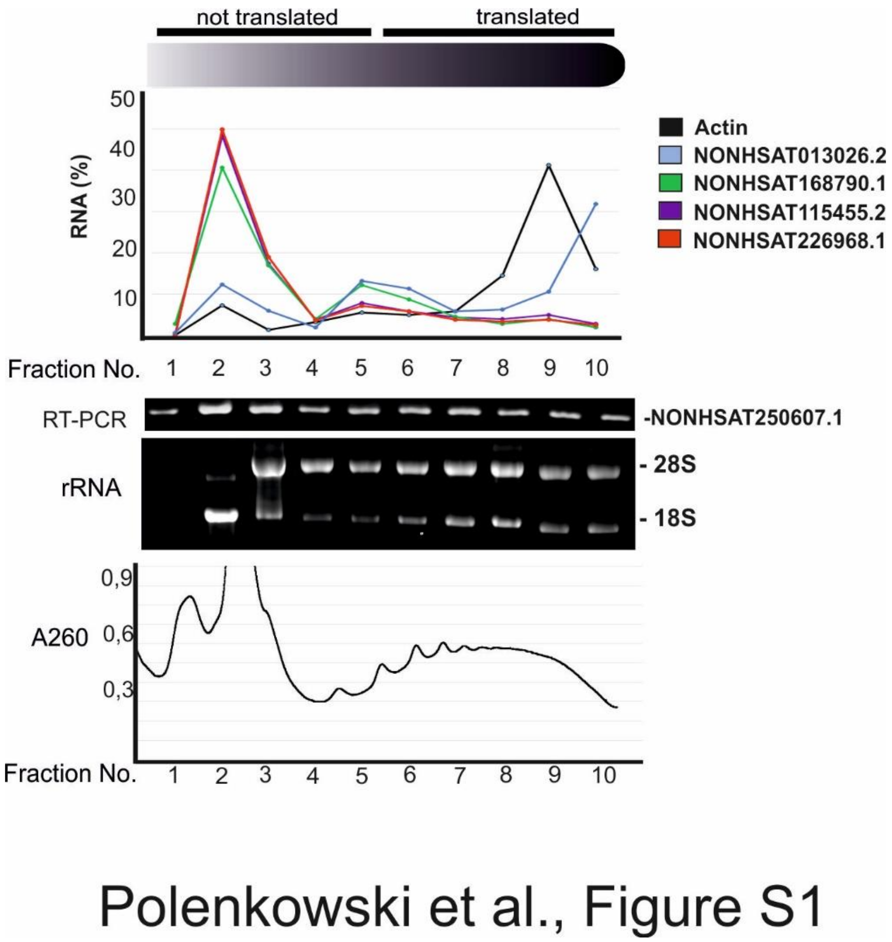

Figure S1. Replicate of polysome profiling experiment.

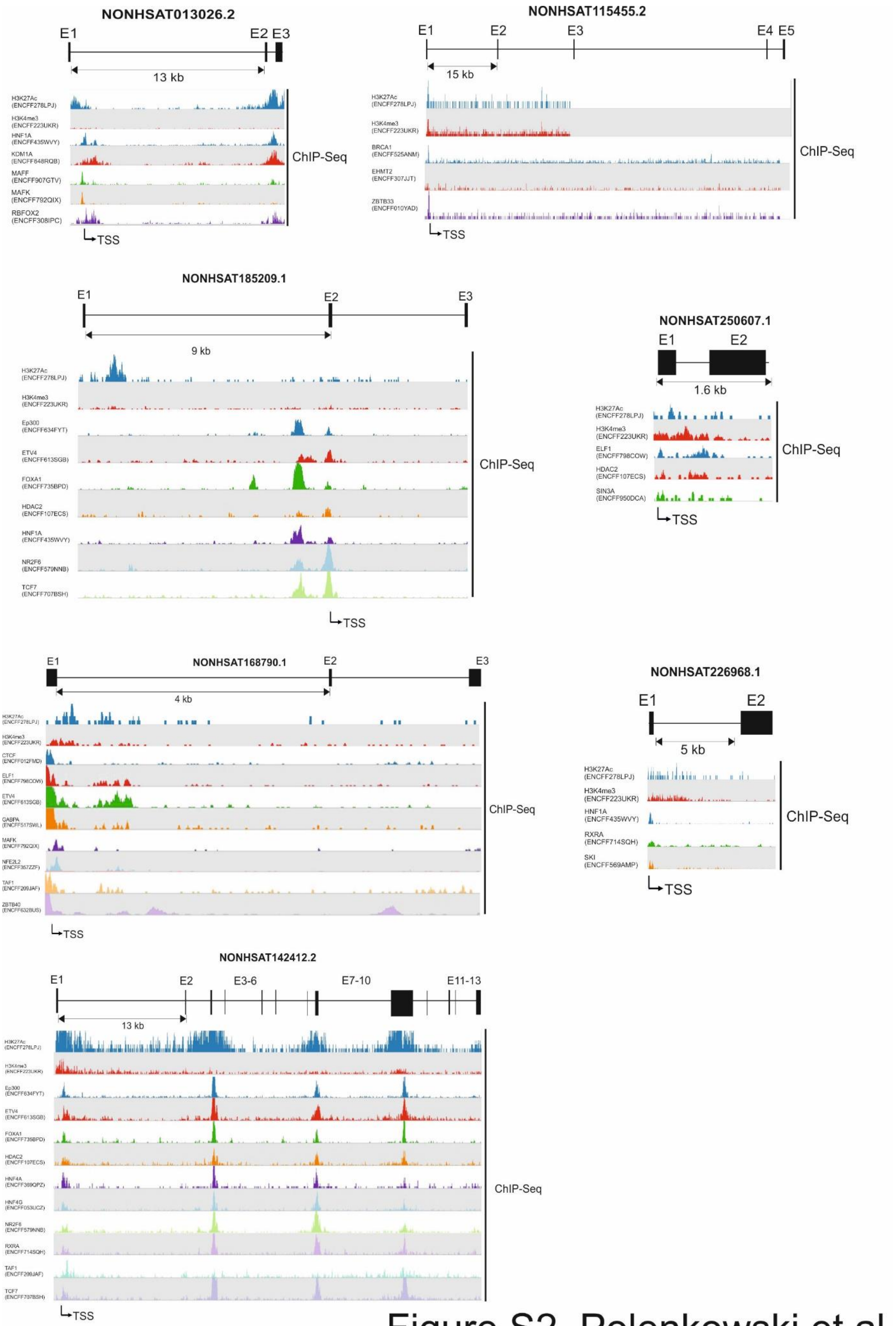

Figure S2, Polenkowski et al.

Figure S2. ChIP-seq of H3K4me3 and H3K27Ac and transcription factors that potentially initiate gene transcription of HCC-specific lncRNAs in HepG2 cells.

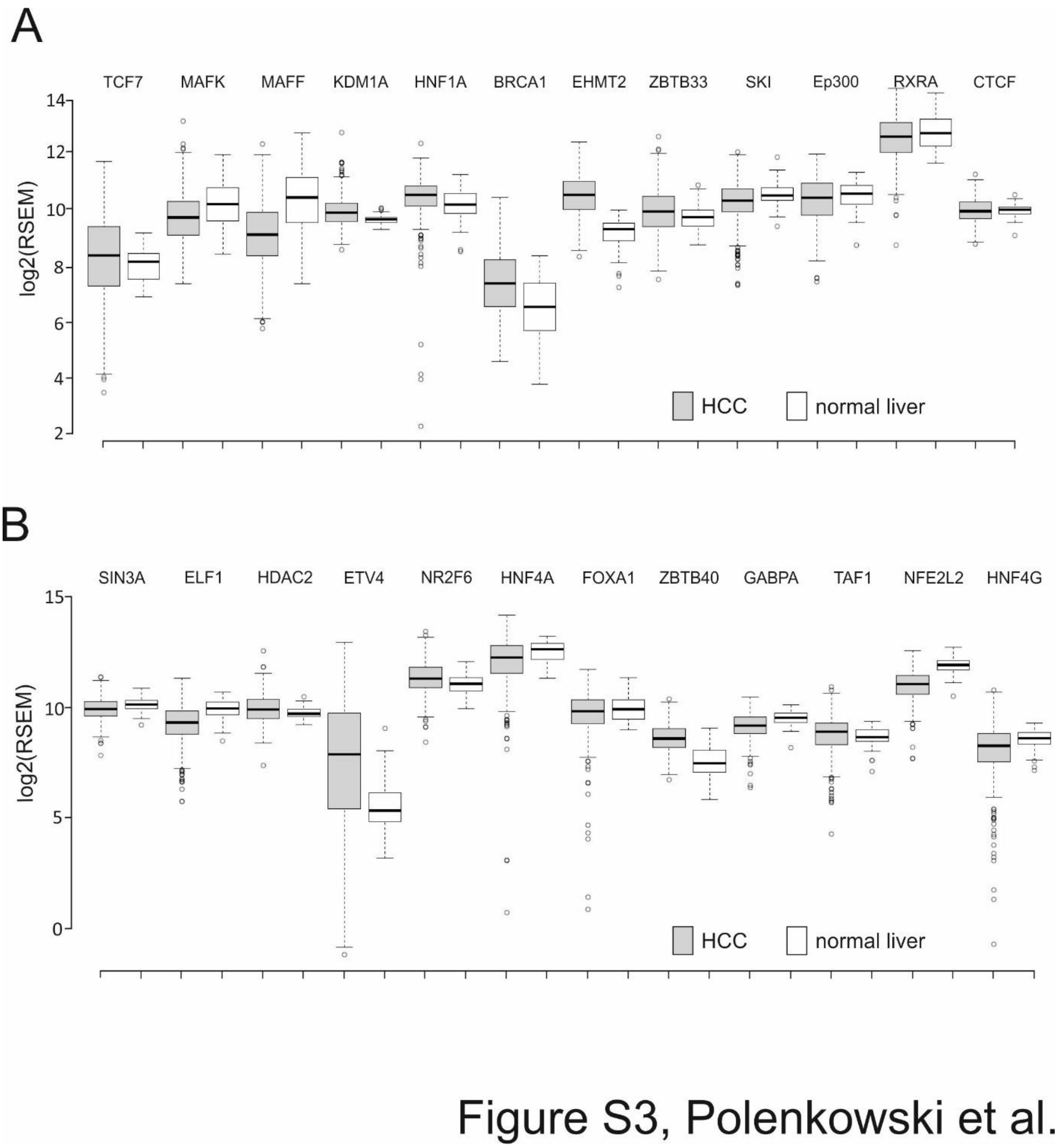

Figure S3. RNA expression level of transcription factors that potentially initiate gene transcription of HCC-specific lncRNAs in normal liver and primary HCC retrieved from TCGA data.

References

1. Songyang, Z.; Cantley, L. C., Recognition and specificity in protein tyrosine kinase-mediated signalling. *Trends in biochemical sciences* **1995,** 20, (11), 470-5.

2. Asante-Appiah, E.; Ball, K.; Bateman, K.; Skorey, K.; Friesen, R.; Desponts, C.; Payette, P.; Bayly, C.; Zamboni, R.; Scapin, G.; Ramachandran, C.; Kennedy, B. P., The YRD motif is a major determinant of substrate and inhibitor specificity in T-cell protein-tyrosine phosphatase. *The Journal of biological chemistry* **2001,** 276, (28), 26036-43.
3. Schwartz, D.; Gygi, S. P., An iterative statistical approach to the identification of protein phosphorylation motifs from large-scale data sets. *Nature biotechnology* **2005,** 23, (11), 1391-8.
4. Onorato, J. J.; Palczewski, K.; Regan, J. W.; Caron, M. G.; Lefkowitz, R. J.; Benovic, J. L., Role of acidic amino acids in peptide substrates of the beta-adrenergic receptor kinase and rhodopsin kinase. *Biochemistry* **1991,** 30, (21), 5118-25.
5. Lees-Miller, S. P.; Anderson, C. W., The DNA-activated protein kinase, DNA-PK: a potential coordinator of nuclear events. *Cancer cells* **1991,** 3, (9), 341-6.
6. Roach, P. J., Multisite and hierarchal protein phosphorylation. *The Journal of biological chemistry* **1991,** 266, (22), 14139-42.
7. Marin, O.; Bustos, V. H.; Cesaro, L.; Meggio, F.; Pagano, M. A.; Antonelli, M.; Allende, C. C.; Pinna, L. A.; Allende, J. E., A noncanonical sequence phosphorylated by casein kinase 1 in beta-catenin may play a role in casein kinase 1 targeting of important signaling proteins. *Proceedings of the National Academy of Sciences of the United States of America* **2003,** 100, (18), 10193-200.
8. Watanabe, F.; Teraoka, H.; Iijima, S.; Mimori, T.; Tsukada, K., Molecular properties, substrate specificity and regulation of DNA-dependent protein kinase from Raji Burkitt's lymphoma cells. *Biochimica et biophysica acta* **1994,** 1223, (2), 255-60.
9. Luo, M.; Reyna, S.; Wang, L.; Yi, Z.; Carroll, C.; Dong, L. Q.; Langlais, P.; Weintraub, S. T.; Mandarino, L. J., Identification of insulin receptor substrate 1 serine/threonine phosphorylation sites using mass spectrometry analysis: regulatory role of serine 1223. *Endocrinology* **2005,** 146, (10), 4410-6.
